# Supplementary material for: Fine-mapping analysis including over 254,000 East Asian and European descendants identifies 136 putative colorectal cancer susceptibility genes
Source: Nat Commun. 2024 Apr 26;15:3557. doi: 10.1038/s41467-024-47399-x (PMC11053150; doi:10.1038/s41467-024-47399-x)
Supplement: Supplementary file 1 — Supplementary Information [file 41467_2024_47399_MOESM1_ESM.pdf]

Supplementary Note

GWAS datasets

We analyzed GWAS data from previously published GWAS [1–5]. In a recent trans-ancestry meta-analysis of GWAS, we grouped participants into analytical units by study or genotyping platform [6]. Studies that contributed to more than one prior GWAS were analyzed only once in the meta-analysis analysis. In total, there were 31 analytical units (17 from European descent populations and 14 from Asian descent populations), totaling 100,204 CRC cases and 154,587 controls. Comprehensive details on the subjects, genotyping, and standard quality control (QC) procedures have been previously reported and are summarized in Fernandez-Rozadilla et al. Nat Genet. 2023 [6]. The summary statistics from these 31 GWAS datasets or that of each population were meta-analyzed under the fixed-effects inverse variance weighted model implemented in METAL [7]. Only variants with an imputation quality score (info/R2) > 0.4, minor allele frequency > 0.005, and present in at least half of included datasets were included in the meta-analysis. The I^2 statistic was calculated to quantify heterogeneity between studies and variants with $I^2 > 65\%$ were excluded. None strong evidence of genomic inflation were observed for each analytical with genomic inflation factor (λ) ranged from 0.95 to 1.28. For the meta-analysis, the λ value was 1.30 (λ1000 = 1.01). As reference for LD estimation, we made use of genotyping data from 6,684 unrelated East Asian samples genotyped with MEGA array (interindividual genetic relationships < 0.025, 453 from Aichi1, 162 from HCES1, 1,764 from HCES2, 832 from Korea_NCC, 312 from Korea_NCC2, 405 from Korea_seoul, 1,833 from Shanghai4, 70 from SBCS1, 426 from SBCS2, and 427 from the lung cancer Asian study cohort) [4] and that from 503 European samples in the 1000 Genome project. All study protocols were approved by the relevant Institutional Review Boards, and informed consent was obtained from all study participants in accordance with the Helsinki accord.

Gene expression and methylation profiles

BarcUVa-Seq: The BarcUVa-Seq (University of Barcelona and University of Virginia RNA sequencing project) gene expression dataset was based on 423 individuals of European ancestry (152 male and 271 female, mean age = 59.8) who received an indication for

colonoscopy at the Bellvitge University Hospital (Hospitalet de Llobregat, Catalonia, Spain) or at the Viladecans Hospital (Viladecans, Catalonia, Spain). Included subjects had no personal history of CRC and underwent negative screening colonoscopies [8,9]. Biopsies from the proximal, transverse, and distal colon mucosa were obtained together with peripheral blood samples. Total RNA was extracted using mirVana kits (Thermo Fisher Scientific) without miRNA enrichment. Libraries were prepared using Illumina TruSeq Stranded Total RNA Library Prep Gold kits, which include Ribo-Zero Plus rRNA Depletion kits for depletion of ribosomal RNA. Paired-end RNA sequencing was performed using an Illumina HiSeq 2500 sequencer in High Output mode. Genotyping of around 400,000 SNPs using DNA isolated from peripheral blood samples was performed using the Illumina OncoArray BeadChip. The study protocol was approved by the Bellvitge University Hospital Ethics Committee (PR073/11 and PR286/15).

Colonomics: The Colonomics gene expression dataset was based on initial 100 CRC patients and 50 healthy donors of European-ancestry (mean age: 63 for patients and 71 for healthy donors; sex: 28% females among patients and 50% females among healthy donors) [10,11]. All the CRC patients who were diagnosed in stage II were surgically treated and had not received adjuvant chemotherapy. Adjacent normal mucosa samples from patients were obtained from the proximal tumor resection margin with a minimum distance of 10 cm. Healthy donors underwent a colonoscopy indicated for screening or symptoms with no evidence of lesions in the colon or rectum and biopsies from left and right colon mucosa were obtained. All subjects provided written informed consent to participate in the study and the ethics committee of the hospital cleared the protocol. The final sample after quality control of the data was 144 including 97 from patients and 47 from health donors. Total RNA was isolated from samples with a miRCURY™ RNA Isolation Kit (Exiqon) and quantified by NanoDrop® ND-1000 Spectrophotometer (Nanodrop technologies, Wilmington, DE). The quality of RNA samples was evaluated using the RNA 6000 Nano Assay (Agilent Technologies, Santa Clara, CA). RNA extracted from each sample was hybridized in Affymetrix chips Human Genome U219.

The Colonomics methylation profiles were generated on 132 mucosa samples including 95 adjacent to tumors from CRC patients and 37 from healthy donors described above [12]. DNA was extracted with phenol-chloroform and quantified by Nanodrop (Thermo Scientific, Wilmington,

DE). Bisulfite conversion of DNA (200-500 ng) was performed using Illumina Infinium Assays (EZ DNA methylation kit. Zymo Research. Cat. No. D5004), and samples were profiled using Illumina Infinium HumanMethylation450 BeadChips. DNA genotyping on genomic DNA from mucosa tissue was performed using Affymetrix Genome-Wide Human SNP 6.0 array (Affymetrix, Santa Clara, CA).

ACCC: The RNA-seq dataset was based on 364 CRC patients of East Asian-ancestry (mean age = 62.8) who participated in the ACCC [13]. Total RNA was isolated from formalin-fixed paraffin-embedded (FFPE normal colon tissue adjacent to tumor using Qiagen's miRNeasy FFPE Kit. The quantity and quality of the RNA samples extracted from tumor tissue FFPE sections were evaluated using Nanodrop (E260, E260/E280 ratio, spectrum 220–320 nm) and were separated on an Agilent BioAnalyzer. Both Ribo-Zero and RNase H were used for rRNA depletion. Illumina TruSeq RNA sample Prep Kit v2 was used to prepare a sequencing library, and HiSeq 2000 was used for paired-end sequencing.

GTEx v8: The gene expression and methylation data were generated from 368 and 189 samples derived from the Transverse colons, respectively [14,15]. eQTL and mQTL data were obtained from GTEx website (https://www.gtexportal.org/home/downloads/adult-gtex/qtl). No participants were compensated to participate in the presented studies.

Supplementary Figures

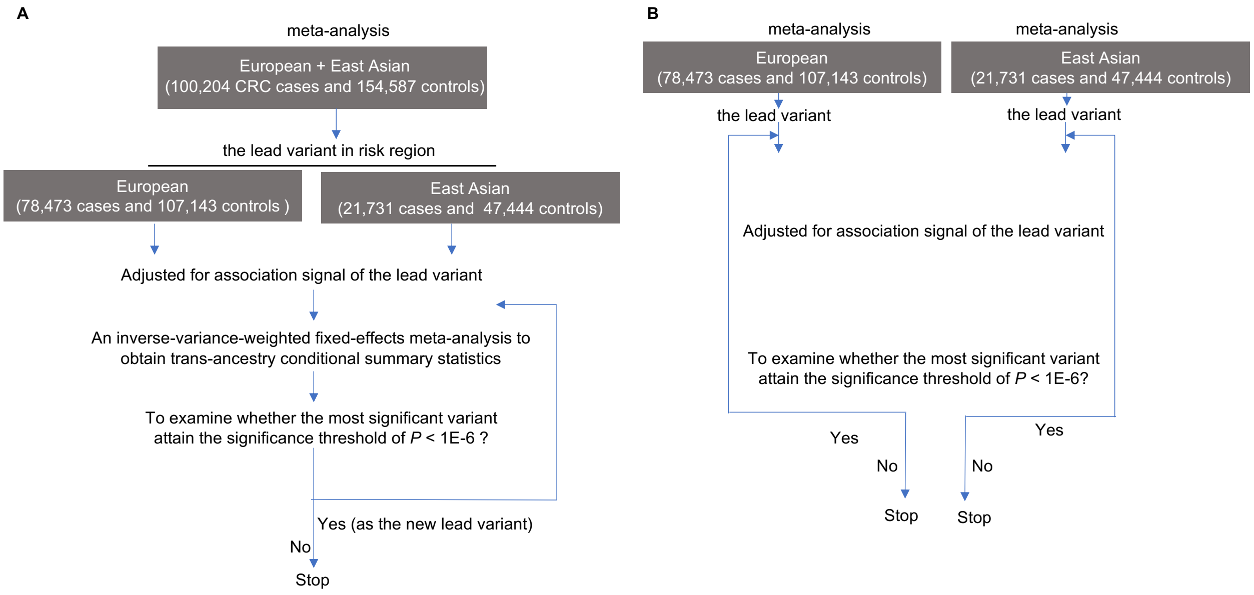

Supplementary Figure 1. **Illustration of stepwise conditional analysis using GCTA-COJO. A** The stepwise conditional analysis for summary statistics from the trans-ancestral meta-analysis. We included common variants (MAF> 0.01) with associations at $P < 0.05$ in both populations. To account for differences in the LD structure, we conducted conditional analysis in each population for each fine-mapping region, conditioning on the most significant association from the trans-ancestral summary statistics. We then meta-analyzed the conditioned results using the fixed-effects inverse variance weighted model with METAL. We applied the conditional $P < 1 \times 10^{-6}$ to define the independent signal. We added any additional variant that remained an independent signal at the conditional $P < 1 \times 10^{-6}$ to the conditional set. We then repeated the conditional analysis until no more variants met the significance threshold. **B** The stepwise conditional analysis for ancestry-specific summary statistics. We included common variants (MAF > 0.01) with associations at $P < 1 \times 10^{-4}$ in each population. Similar to the trans-ancestral analysis, we added any additional variant that remained an independent signal at the conditional $P < 1 \times 10^{-6}$ to the conditional set and repeated the conditional analysis until no more variants met the significance threshold.

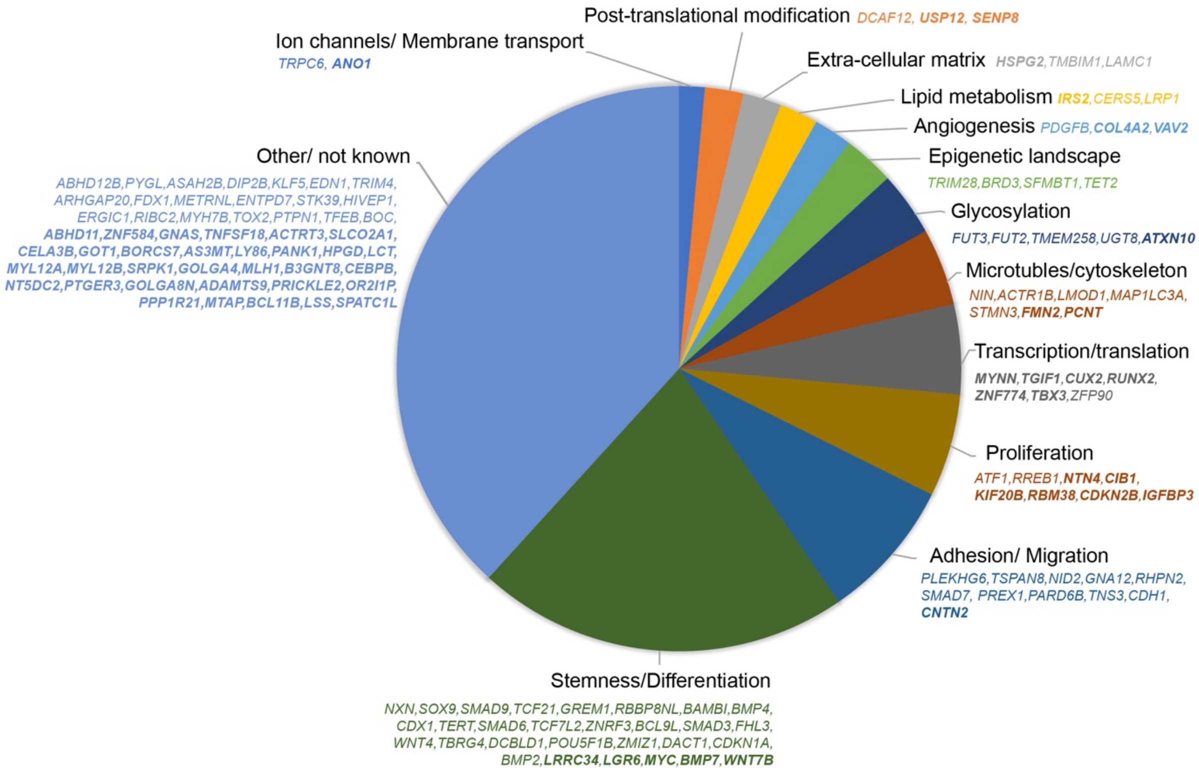

Supplementary Figure 2. **Cellular function of each credible target gene.** Pie chart showing the allocation of each credible target gene to cellular processes described in *ref.6*.

Reference

1. Huyghe, J. R. *et al.* Discovery of common and rare genetic risk variants for colorectal cancer. *Nat. Genet.* **51**, 76–87 (2019).
2. Law, P. J. *et al.* Association analyses identify 31 new risk loci for colorectal cancer susceptibility. *Nat. Commun.* **10**, 2154 (2019).
3. Lu, Y. *et al.* Identification of novel loci and new risk variant in known loci for colorectal cancer risk in East Asians. *Cancer Epidemiol. Biomarkers Prev.* **29**, 477–486 (2020).
4. Lu, Y. *et al.* Large-scale genome-wide association study of East Asians identifies loci associated with risk for colorectal cancer. *Gastroenterology* **156**, 1455–1466 (2019).
5. Schmit, S. L. *et al.* Novel common genetic susceptibility loci for colorectal cancer. *J. Natl. Cancer Inst.* **111**, 146–157 (2019).
6. Fernandez-Rozadilla, C. *et al.* Deciphering colorectal cancer genetics through multi-omic analysis of 100,204 cases and 154,587 controls of European and east Asian ancestries. *Nat. Genet.* **55**, 519–520 (2023).

7. Willer, C. J., Li, Y. & Abecasis, G. R. METAL: fast and efficient meta-analysis of genomewide association scans. *Bioinformatics* **26**, 2190–2191 (2010).
8. Díez-Obrero, V. *et al.* Genetic effects on transcriptome profiles in colon epithelium provide functional insights for genetic risk loci. *Cell. Mol. Gastroenterol. Hepatol.* **12**, 181–197 (2021).
9. Dampier, C. H. *et al.* Oncogenic features in histologically normal mucosa: Novel insights into field effect from a mega-analysis of colorectal transcriptomes. *Clin. Transl. Gastroenterol.* **11**, e00210 (2020).
10. Sanz-Pamplona, R. *et al.* Aberrant gene expression in mucosa adjacent to tumor reveals a molecular crosstalk in colon cancer. *Mol. Cancer* **13**, 46 (2014).
11. Closa, A. *et al.* Identification of candidate susceptibility genes for colorectal cancer through eQTL analysis. *Carcinogenesis* **35**, 2039–2046 (2014).
12. Díez-Villanueva, A. *et al.* Identifying causal models between genetically regulated methylation patterns and gene expression in healthy colon tissue. *Clin. Epigenetics* **13**, 162 (2021).
13. Chen Z, Song W, Wen W , Devall M, Dampier C. Unveiling Susceptible Transcription Factors and Novel Risk Genes in Colorectal Cancer: Integrating LargeScale Genetic and Omics Data Analysis. *ASHG*.
14. The GTEx Consortium *et al.* The GTEx Consortium atlas of genetic regulatory effects across human tissues. *Science* **369**, 1318–1330 (2020).
15. Oliva, M. *et al.* DNA methylation QTL mapping across diverse human tissues provides molecular links between genetic variation and complex traits. *Nat. Genet.* **55**, 112–122 (2023).
