# Supplementary material for: Fine-mapping analysis including over 254,000 East Asian and European descendants identifies 136 putative colorectal cancer susceptibility genes
Source: Nat Commun. 2024 Apr 26;15:3557. doi: 10.1038/s41467-024-47399-x (PMC11053150; doi:10.1038/s41467-024-47399-x)
Supplement: Supplementary file 3 — Description of Additional Supplementary Files [file 41467_2024_47399_MOESM3_ESM.pdf]

Description of Additional Supplementary Files

Supplementary Data 1. Colorectal cancer GWAS included in the fine-mapping analysis.

Supplementary Data 2. List of 143 fine-mapping genomic regions containing 205 GWAS-reported genetic variants for colorectal cancer risk.

Supplementary Data 3. CRC independent association signals identified through stepwise conditional analysis in trans-ancestry analyses.

Supplementary Data 4. CRC independent association signals identified through stepwise conditional analysis in European-ancestry populations.

Supplementary Data 5. CRC independent association signals identified through stepwise conditional analysis in East Asian-ancestry populations.

Supplementary Data 6. List of 5,741 CCVs identified in this study for CRC independent association signals.

Supplementary Data 7. Comparisons of CCV size for 138 independent association signals identified in both trans-ancestry and European-ancestry specific analyses.

Supplementary Data 8. Publicly available data used in this study for epigenomic characterization of CCVs.

Supplementary Data 9. Scoring hierarchy in in silico prediction approach (a modified INQUSIT).

Supplementary Data 10. List of 72 predicted target genes likely regulated by distal CCVs.

Supplementary Data 11. List of 48 predicted target genes likely regulated by proximal CCVs.

Supplementary Data 12. List of 19 predicted target genes for CCVs in coding region.

Supplementary Data 13. Significant cis-eQTLs for independent association signals.

Supplementary Data 14. Significant cis-mQTLs for independent association signals.

Supplementary Data 15. Significant colocalization between CCVs and eQTL gene identified in

trans-ancestral independent association signals.

Supplementary Data 16. Significant colocalization for CCVs and eQTL gene identified in European independent association signals.

Supplementary Data 17. Significant colocalization for CCVs and eQTL gene identified in East Asian independent association signals.

Supplementary Data 18. Significant colocalization for CCVs and mQTL gene identified in trans-ancestral independent association signals.

Supplementary Data 19. Significant colocalization for CCVs and mQTL gene identified in European independent association signals.

Supplementary Data 20. List of 288 non-pseudogenes targets of CCVs supported by either functional genomic data or colocalization analysis.

Supplementary Data 21. Differential gene expression analysis for the 136 credible target genes across nine cell types in colon tissue.

Supplementary Data 22. Pathways significantly enriched in the 136 credible target genes of CCVs.
