# Supplementary material for: Fine-mapping analysis including over 254,000 East Asian and European descendants identifies 136 putative colorectal cancer susceptibility genes
Source: Nat Commun. 2024 Apr 26;15:3557. doi: 10.1038/s41467-024-47399-x (PMC11053150; doi:10.1038/s41467-024-47399-x)
Supplement: Supplementary file 5 — Reporting Summary [file 41467_2024_47399_MOESM5_ESM.pdf]

Corresponding author(s): Wei Zheng

Last updated by author(s): 02/10/2024

# Reporting Summary

Nature Portfolio wishes to improve the reproducibility of the work that we publish. This form provides structure for consistency and transparency in reporting. For further information on Nature Portfolio policies, see our Editorial Policies and the Editorial Policy Checklist.

Please do not complete any field with "not applicable" or n/a. Refer to the help text for what text to use if an item is not relevant to your study.
For final submission: please carefully check your responses for accuracy; you will not be able to make changes later.

## Statistics

For all statistical analyses, confirm that the following items are present in the figure legend, table legend, main text, or Methods section.

| n/a | Confirmed | |
|---|---|---|
| ☐ | ☒ | The exact sample size (*n*) for each experimental group/condition, given as a discrete number and unit of measurement |
| ☐ | ☒ | A statement on whether measurements were taken from distinct samples or whether the same sample was measured repeatedly |
| ☐ | ☒ | The statistical test(s) used AND whether they are one- or two-sided<br>*Only common tests should be described solely by name; describe more complex techniques in the Methods section.* |
| ☐ | ☒ | A description of all covariates tested |
| ☐ | ☒ | A description of any assumptions or corrections, such as tests of normality and adjustment for multiple comparisons |
| ☐ | ☒ | A full description of the statistical parameters including central tendency (e.g. means) or other basic estimates (e.g. regression coefficient) AND variation (e.g. standard deviation) or associated estimates of uncertainty (e.g. confidence intervals) |
| ☐ | ☒ | For null hypothesis testing, the test statistic (e.g. *F*, *t*, *r*) with confidence intervals, effect sizes, degrees of freedom and *P* value noted<br>*Give P values as exact values whenever suitable.* |
| ☒ | ☐ | For Bayesian analysis, information on the choice of priors and Markov chain Monte Carlo settings |
| ☒ | ☐ | For hierarchical and complex designs, identification of the appropriate level for tests and full reporting of outcomes |
| ☐ | ☒ | Estimates of effect sizes (e.g. Cohen's *d*, Pearson's *r*), indicating how they were calculated |

*Our web collection on statistics for biologists contains articles on many of the points above.*

## Software and code

Policy information about availability of computer code

| | |
|---|---|
| Data collection | No software was used for data collection. |
| Data analysis | bedtools (https://bedtools.readthedocs.io/) was used to aggregate CRC GWAS risk variants to fine-mapping regions. GCTA-COJO (https://cnsgenomics.com/software/gcta) was used to perform conditional analysis to identify independent association signals. METAL (https://genome.sph.umich.edu/wiki/METAL_Documentation) was used to combine conditioned results in two populations. LDlinkR (https://github.com/CBIIT/LDlinkR) was used to examine linkage disequilibrium between variants. STAR (v2.5.4) was used to map sequencing reads to human reference genome. RNA-SeQC (v2.3.5) was used to quality control of RNA-seq samples and quantify gene-level expression. MACS (https://github.com/macs3-project/MACS) was used to convert coverage tracks Bigwig (bw) files to bedGraph files for ChIP-seq data and then identify binding peaks. Variant Effect Predictor (VEP) (https://useast.ensembl.org/info/docs/tools/vep/index.html) was used to conduct variant annotation of credible causal variants (CCVs). Enrichr (https://maayanlab.cloud/Enrichr/) was used to perform gene pathway analysis. ANNOVAR (https://annovar.openbioinformatics.org/) was used to annotate all variants in the UKBB WES 200K cohort. Custom code (https://github.com/zhishanchen/CRC_Finemapping) was used in this study. |

For manuscripts utilizing custom algorithms or software that are central to the research but not yet described in published literature, software must be made available to editors and reviewers. We strongly encourage code deposition in a community repository (e.g. GitHub). See the Nature Portfolio guidelines for submitting code & software for further information.

# Data

Policy information about availability of data

All manuscripts must include a data availability statement. This statement should provide the following information, where applicable:

- Accession codes, unique identifiers, or web links for publicly available datasets
- A description of any restrictions on data availability
- For clinical datasets or third party data, please ensure that the statement adheres to our policy

The GWAS summary statistics are available at the GWAS catalog under accession number GCST90129505 (https://www.ebi.ac.uk/gwas/studies/GCST90129505). The RNA-seq data and genotype data of subjects of East Asian ancestry from the ACCC is being deposited to NCBI database of Genotypes and Phenotypes (dbGaP). All requests to access these data could also be made by contacting Drs. Wei Zheng (wei.zheng@vanderbilt.edu) and Xingyi Guo (xingyi.guo@vumc.org). The data from the Genotype-Tissue Expression (GTEx, version 8) project used in this study are publicly available at the dbGaP under accession number phs000424.v8.p2 (https://www.ncbi.nlm.nih.gov/projects/gap/cgi-bin/study.cgi?study_id=phs000424.v8.p2). The transcriptome and genotype data as well as the sample covariates from the BarcUVa-Seq project can be accessed at the dbGaP under accession number phs003338.v1.p1 (https://www.ncbi.nlm.nih.gov/projects/gap/cgi-bin/study.cgi?study_id=phs003338.v1.p1). The access to data from the Colonomics project could be requested by submission of an inquiry to Dr. Victor Moreno (v.moreno@iconcologia.net). The CRC-relevant epigenome and functional genomic data were obtained from the NCBI's Gene Expression Omnibus database (GEO) under accession numbers: GSE133928 (https://www.ncbi.nlm.nih.gov/geo/query/acc.cgi?acc=GSE133928), GSE136889 (https://www.ncbi.nlm.nih.gov/geo/query/acc.cgi?acc=GSE136889), and GSE156613 (https://www.ncbi.nlm.nih.gov/geo/query/acc.cgi?acc=GSE156613). Enhancer-promoter interaction data were obtained from the ENdb database (https://bio.liclab.net/ENdb/), 4Dgenome (https://4dgenome.research.chop.edu/), FANTOM5 (https://fantom.gsc.riken.jp/5/), EnhancerAtlas 2.0 (http://www.enhanceratlas.org/) and Super-enhancers (https://bio.liclab.net/sedb/ and https://www.cell.com/fulltext/S0092-8674(13)01227-0#supplementaryMaterial). Single-cell RNA-sequencing datasets from colon tissues of 31 individuals were obtained from the Colorectal Molecular Atlas Project (COLON MAP). Whole exome sequencing data from 137,104 individuals of European ancestry were obtained from the UK Biobank (https://www.ukbiobank.ac.uk/).

# Research involving human participants, their data, or biological material

Policy information about studies with human participants or human data. See also policy information about sex, gender (identity/presentation), and sexual orientation and race, ethnicity and racism.

| | |
|---|---|
| Reporting on sex and gender | The term "sex" was used in this study. Sex was used as a covariate in association analyses including eQTL and gene burden test. |
| Reporting on race, ethnicity, or other socially relevant groupings | The analyses were conducted using individuals of European ancestry and East Asian ancestry. |
| Population characteristics | Unrelated individuals of European ancestry and East Asian ancestry were included our analysis. |
| Recruitment | We analyzed genomic data from previously published GWAS. Details of the participant selection were previously reported and summarized in previous studies (PMID: 36539618, PMID: 31089142, PMID: 30510241, PMID: 31826910). Descriptions of study participants in the GTEx Project, the BarcUVa-Seq project, and the Colonomics project were described in detail in previous studies (PMID: 32913098, PMID: 33601062 and PMID: 36182938). Details for individuals from UK Biobank can be found at https://www.ukbiobank.ac.uk/. |
| Ethics oversight | All study protocols were approved by the relevant Institutional Review Boards, and informed consent was obtained from all study participants in accordance with the Helsinki accord. |

Note that full information on the approval of the study protocol must also be provided in the manuscript.

# Field-specific reporting

Please select the one below that is the best fit for your research. If you are not sure, read the appropriate sections before making your selection.

☒ Life sciences ☐ Behavioural & social sciences ☐ Ecological, evolutionary & environmental sciences

For a reference copy of the document with all sections, see nature.com/documents/nr-reporting-summary-flat.pdf

# Life sciences study design

All studies must disclose on these points even when the disclosure is negative.

| | |
|---|---|
| Sample size | All available GWAS data from colorectal cancer cases and controls derived from studies of > 1000 individuals of European or East Asian ancestry populations were included. For RNA-seq data for eQTL and CpG methylation data for mQTL, we collected datasets as many as available. All available whole exome sequencing data from colorectal cancer cases and controls of European ancestry from UK Biobank (UKBB) was included. |
| Data exclusions | The summary statistic data of GWAS included variants with an imputation quality score (info/R2) >0.4 and seen in at least 15 analytical units. The I2 statistic was calculated to quantify heterogeneity between study heterogeneity and variants with I2 >65% were excluded. The fine-mapping analysis and downstream analyses excluded the genomic risk regions on Xp22.2. For the trans-ancestry condition analysis, the analysis included variants with MAF >0.01 and associations with CRC risk at P< 0.05 in both ancestry populations. For the ancestry-specific conditional analysis, we included variants with MAF >0.01 and association with CRC risk at P > 1E−4. For the RNA-seq data from individuals of East Asian-ancestry, samples that met any of the following criteria were removed: 1) <10 million mapped reads; 2) read mapping rate < 0.2; 3) intergenic mapping rate > 0.4; 4) base mismatch rate > 0.01 for read mate 1 or > 0.02 for read mate 2; and 5) rRNA mapping rate > 0.3. |
| Replication | Replication was not assessed specifically, but was inherent within the GWAS meta-analysis under a fixed-effects inverse-variance weighted model using METAL. In the eQTL and mQTL analysis, the QTL results were combined by the meta-analysis for independent singals with QTL associations in multiple datasets. |
| Randomizatio | The study is observational, and therefore randomization is not necessary. |
| Blinding | The study is observational, and therefore no blinding is necessary. |

# Reporting for specific materials, systems and methods

We require information from authors about some types of materials, experimental systems and methods used in many studies. Here, indicate whether each material, system or method listed is relevant to your study. If you are not sure if a list item applies to your research, read the appropriate section before selecting a response.

## Materials & experimental systems

| n/a | Involved in the study |
|---|---|
| ☒ | ☐ Antibodies |
| ☒ | ☐ Eukaryotic cell lines |
| ☒ | ☐ Palaeontology and archaeology |
| ☒ | ☐ Animals and other organisms |
| ☒ | ☐ Clinical data |
| ☒ | ☐ Dual use research of concern |
| ☒ | ☐ Plants |

## Methods

| n/a | Involved in the study |
|---|---|
| ☒ | ☐ ChIP-seq |
| ☒ | ☐ Flow cytometry |
| ☒ | ☐ MRI-based neuroimaging |
